# Supplementary material for: Drug-induced stress mediates Plasmodium falciparum ring-stage growth arrest and reduces in vitro parasite susceptibility to artemisinin
Source: Microbiol Spectr. 2024 Feb 16;12(4):e03500-23. doi: 10.1128/spectrum.03500-23 (PMC10986542; doi:10.1128/spectrum.03500-23)
Supplement: Supplemental information — Supplemental table and figures. [file spectrum.03500-23-s0001.docx]

**Supplementary Information**

Drug-induced stress mediates *Plasmodium falciparum* ring-stage growth arrest and reduces *in vitro* parasite susceptibility to artemisinin

Lucien Platon , Didier Leroy, David A. Fidock, Didier Ménard

This supplementary information has been provided by the authors to provide readers with additional information about the work.

**Table of contents**

[**1.** **Stress-induced medium mediates delayed growth of *P. falciparum* ring-stages *in vitro*.** 3](#_Toc145923456)

[Table S1. Origin, year of collection and genotypes in Pfkelch13, pfcrt and pfmdr-1 of the parasite lines. 3](#_Toc145923457)

[Figure S1. Schematic representation of the experimental design. 3](#_Toc145923458)

[Figure S2. Killing effect of 200 nM CQ on NF54 CQ-S blood stage parasites. 4](#_Toc145923459)

[**2.** ***In vitro* susceptibility to DHA is reduced in early rings (0-3 hpi) exposed to a stress-induced medium.** 5](#_Toc145923460)

[Figure S3. Schematic representation of the experimental design. 5](#_Toc145923461)

[***3.*** ***In vitro* susceptibility of *P. falciparum* lines to DHA is restored by dilutions of stress-induced medium*.*** 6](#_Toc145923462)

[Figure S4. Schematic representation of the experimental design. 6](#_Toc145923463)

[***4.*** **Ring-stage growth delay and reduced in vitro susceptibility to DHA induced by stress-induced medium are mediated by molecules of different molecular weight, regardless of the *Pfkelch13* genotype*.*** 7](#_Toc145923464)

[Figure S5. Schematic representation of the experimental design. 7](#_Toc145923465)

[**5.** **Mature-stage parasites treated with DHA release molecules into the culture medium that reduce in vitro susceptibility of ring-stage parasites to DHA.** 8](#_Toc145923466)

[Figure S6. Schematic representation of the experimental design. 8](#_Toc145923467)

# **Stress-induced medium mediates delayed growth of *P. falciparum* ring-stages *in vitro*.**

We produced the stress-induced environmental condition by generating a culture medium (named M_NF54_CQ_20h) from an asynchronous NF54 chloroquine-sensitive (CQ-S) parasite line exposed to 200 nM CQ for 20 hours. Cambodian CQ-resistant (CQ-R) *P. falciparum* PL1^C580Y^, PL2^C580Y^ and PL3^WT^ parasite lines (***Table S1***) were cultured and synchronized by exposing predominantly ring-stage cultures to 5% D-Sorbitol (Sigma) for 10 min at 37°C to remove mature parasites. Synchronized ring-stages at 0.5% parasitemia were then exposed to the M_NF54_CQ_20h culture medium or complete RPMI medium (non-exposed, used as control) for 24 hr. After two washing steps with RPMI medium, the parasite lines were cultured for an additional 24 hours in RPMI complete medium (H48). Red blood cells (RBCs) were collected at H24 and H48 to prepare Giemsa-stained blood smears (***Figure S1***). We confirmed the killing effect on NF54 CQ-S blood stages as presented in ***Figure S2*** (***Panel A***. NF54 parasites before addition of 200 nM CQ and ***Panel B***, NF54 parasites after 20 hours incubation with 200 nM CQ).

## ***Table S1. Origin, year of collection and genotypes in Pfkelch13, pfcrt and pfmdr-1 of the parasite lines.***

| **Strain ID** | **Origin** | **Year** | ***Kelch13*** | ***pfcrt*** | ***pfmdr-1*** |
| --- | --- | --- | --- | --- | --- |
| NF54 | Africa | - | Wild type | CVMNKTHFIMCGT | NYSND |
| 3D7 | Africa | - | Wild type | CVMNKTHFIMCGT | NYSND |
| 3D7^C580Y^ | Africa | - | C580Y | CVMNKTHFIMCGT | NYSND |
| PL1^C580Y^ | Pailin | 2010 | C580Y | CVIETTHFIMCGT | NFSND |
| PL2^C580Y^ | Pailin | 2010 | C580Y | CVIETTHFIMCGT | NFSND |
| PL3^WT^ | Pailin | 2010 | Wild type | CVIETTHFIMCGT | NFSND |

Position of amino acid corresponds to codons 72, 73, 74, 76, 93, 97, 145, 218, 343, 350, 353 and 356 for *pfcrt*, and to codons 86, 184, 1034, 1042 and 1246 for *pfmdr-1*.

## ***Figure S1. Schematic representation of the experimental design.***


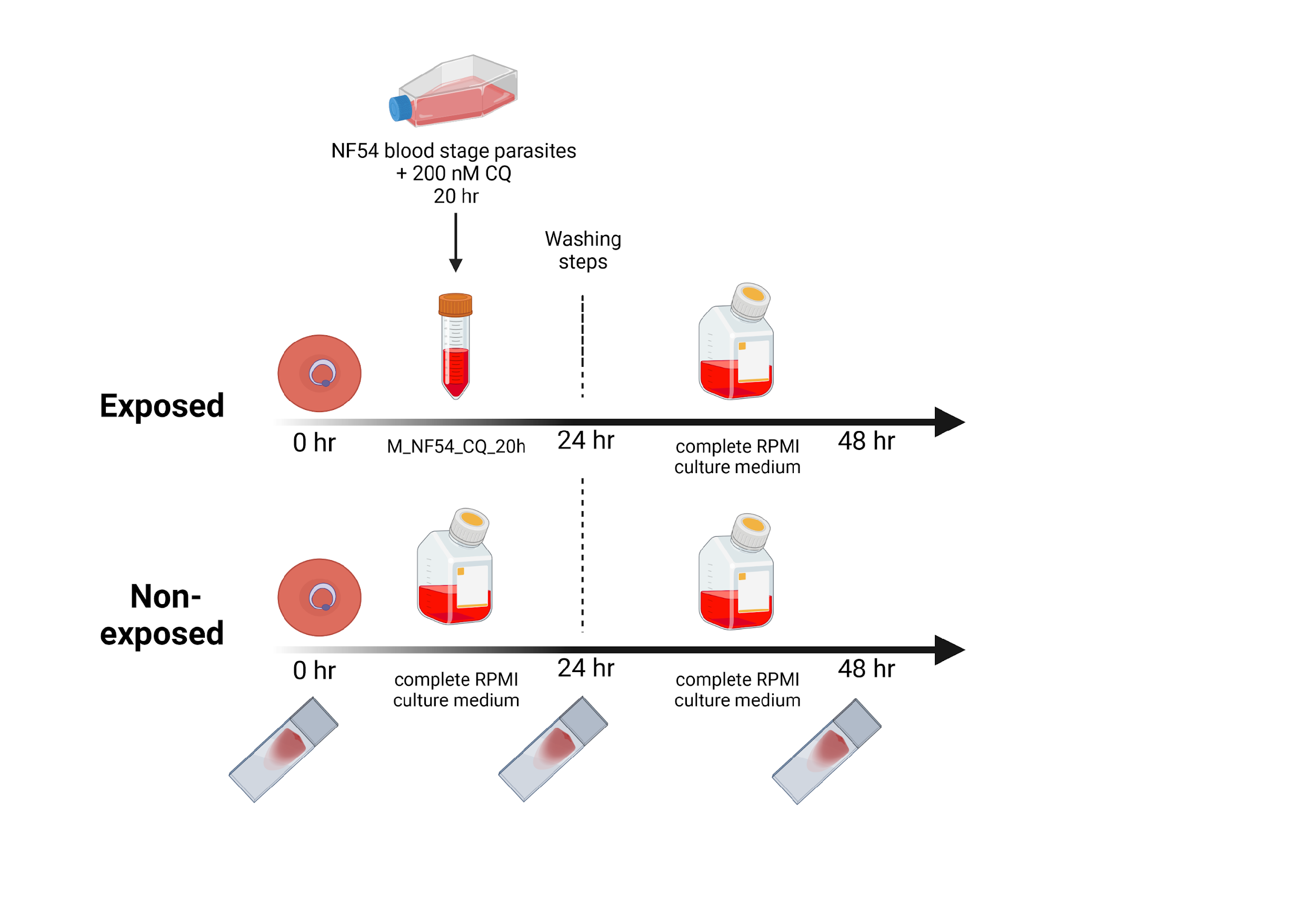


## ***Figure S2. Killing effect of 200 nM CQ on NF54 CQ-S blood stage parasites.***


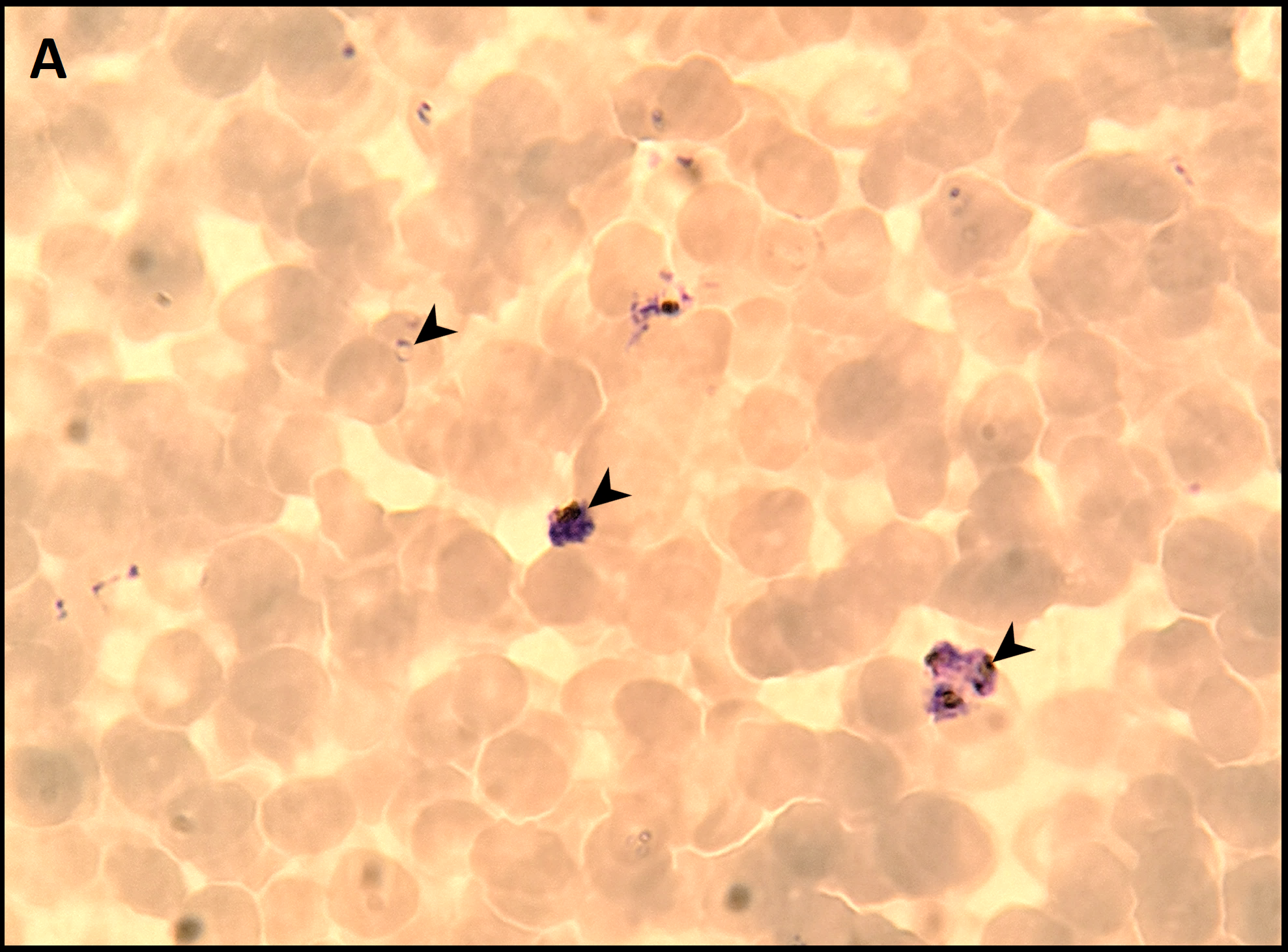

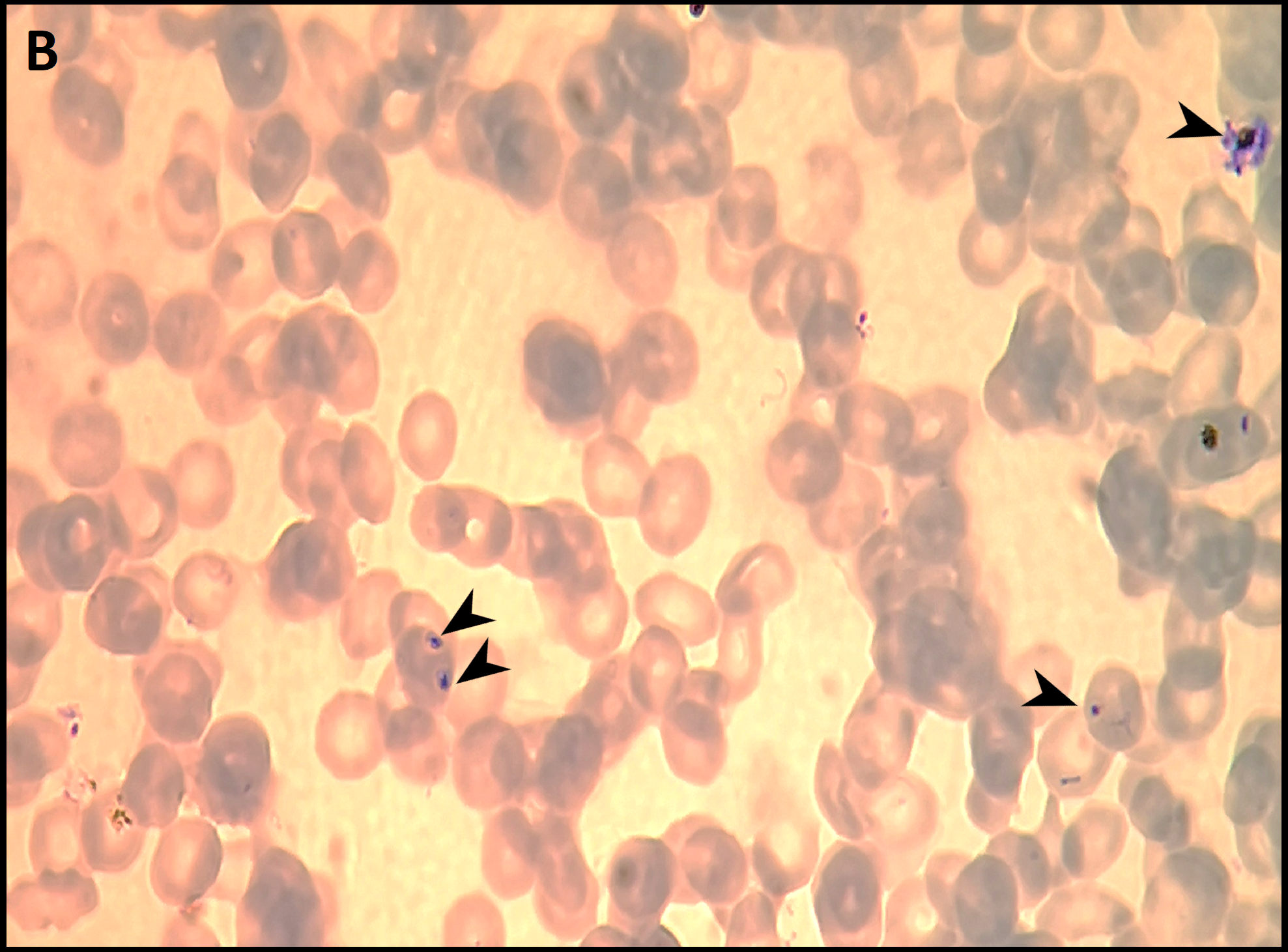


**B**

**A**

**B**

Panel A. Presence of asexual blood-stages (rings, trophozoites and schizonts) (black arrows).

Panel B. Presence of dead or pyknotic parasites (black arrows).

Images were obtained by light microscopy (Giemsa-stained blood smears, magnification x1000).

# ***In vitro* susceptibility to DHA is reduced in early rings (0-3 hpi) exposed to a stress-induced medium.**

## ***Figure S3. Schematic representation of the experimental design.***

***
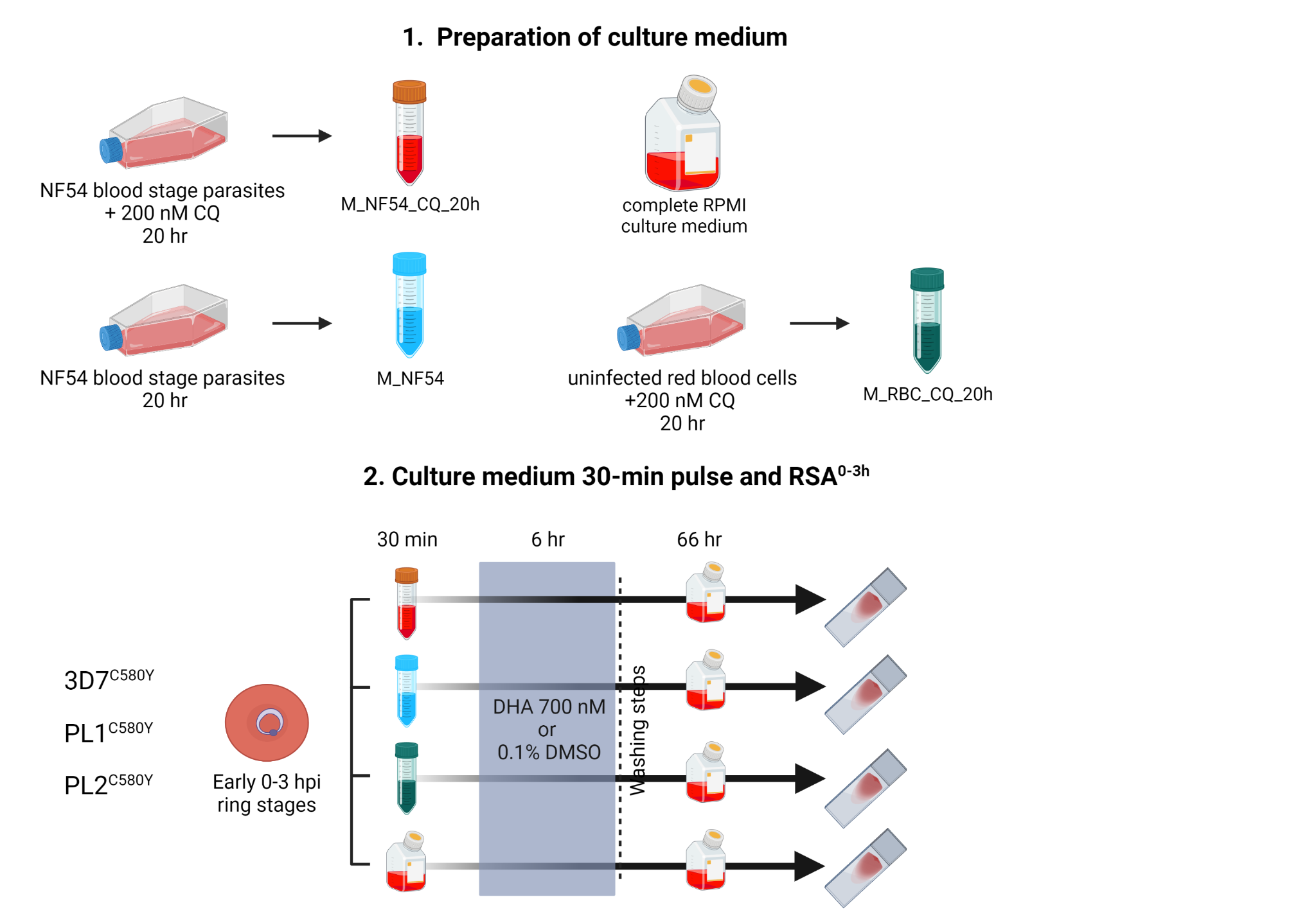
***

Early 0-3 hpi rings of 3D7^C580Y^ (a CRISPR/Cas9-edited line), the PL1^C580Y^ and PL2^C580Y^ lines were pre-exposed to four different culture media for 30 min (RPMI complete medium used as control; M_NF54_CQ20h medium; M_NF54 a culture medium prepared from asynchronous healthy NF54 parasites; and M_RBC_CQ20h, a culture medium prepared from uninfected RBCs exposed to 200 nM CQ for 20 h). The 3D7^C580Y^, PL1^C580Y^ and PL2^C580Y^ lines were then exposed to 700 nM DHA or 0.1% dimethylsulfoxide (DMSO, used as a vehicle control) for 6 hours, washed three times with incomplete RPMI medium to remove the drug, transferred to new wells, and cultured in complete RPMI medium for a further 66 hours. Parasitemia was measured by microscopy at 72 hours. Parasite survival was expressed as the ratio of viable parasites in DHA to DMSO-treated samples. All experiments were performed in triplicate.

# ***In vitro* susceptibility of *P. falciparum* lines to DHA is restored by dilutions of the stress-induced medium*.***

## ***Figure S4. Schematic representation of the experimental design.***


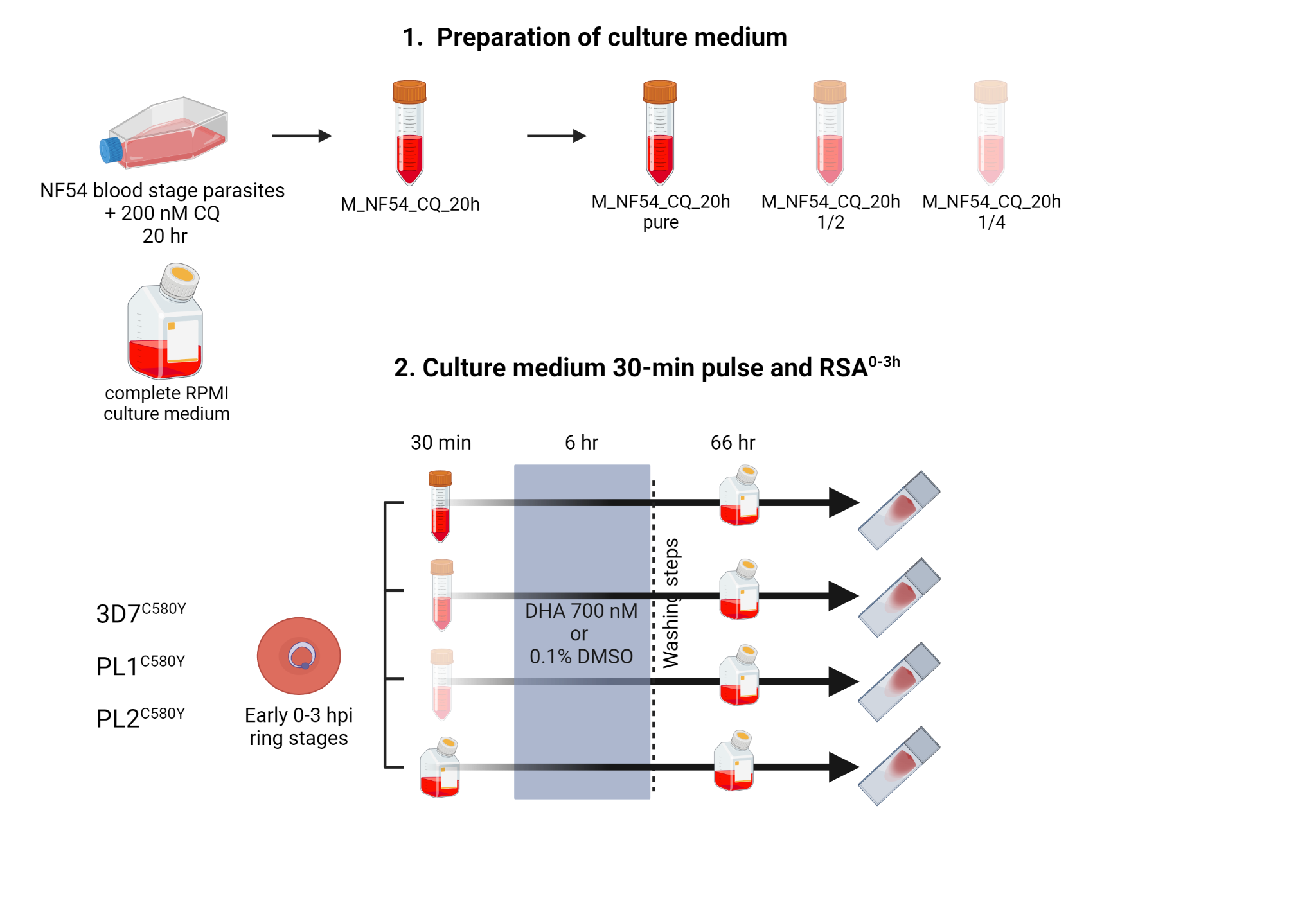


To prepare the dilution of M_NF54_CQ20h medium**,** we used complete RPMI culture medium to dilute M_NF54_CQ20h to ½ (1 volume M_NF54_CQ20h medium and 1 volume of complete RPMI culture medium) and ¼ (1 volume M_NF54_CQ20h medium and 3 volumes of complete RPMI culture medium). Diluted M_NF54_CQ20h medium was kept at 37°C in a water bath until used.

We treated 0-3 hpi ring-stage 3D7^C580Y^, PL1^C580Y^ and PL2^C580Y^ parasites with different dilutions of the M_NF54_CQ20h medium (½ and ¼ dilutions) before assessing their *in vitro* susceptibility to DHA using the RSA^0-3h^. Complete RPMI medium and undiluted M_NF54_CQ20h medium were used as negative and positive controls, respectively. All assays were performed in triplicate.

# **Ring-stage growth delay and reduced in vitro susceptibility to DHA induced by stress-induced medium are mediated by molecules of different molecular weight, regardless of the *Pfkelch13* genotype*.***

## ***Figure S5. Schematic representation of the experimental design.***


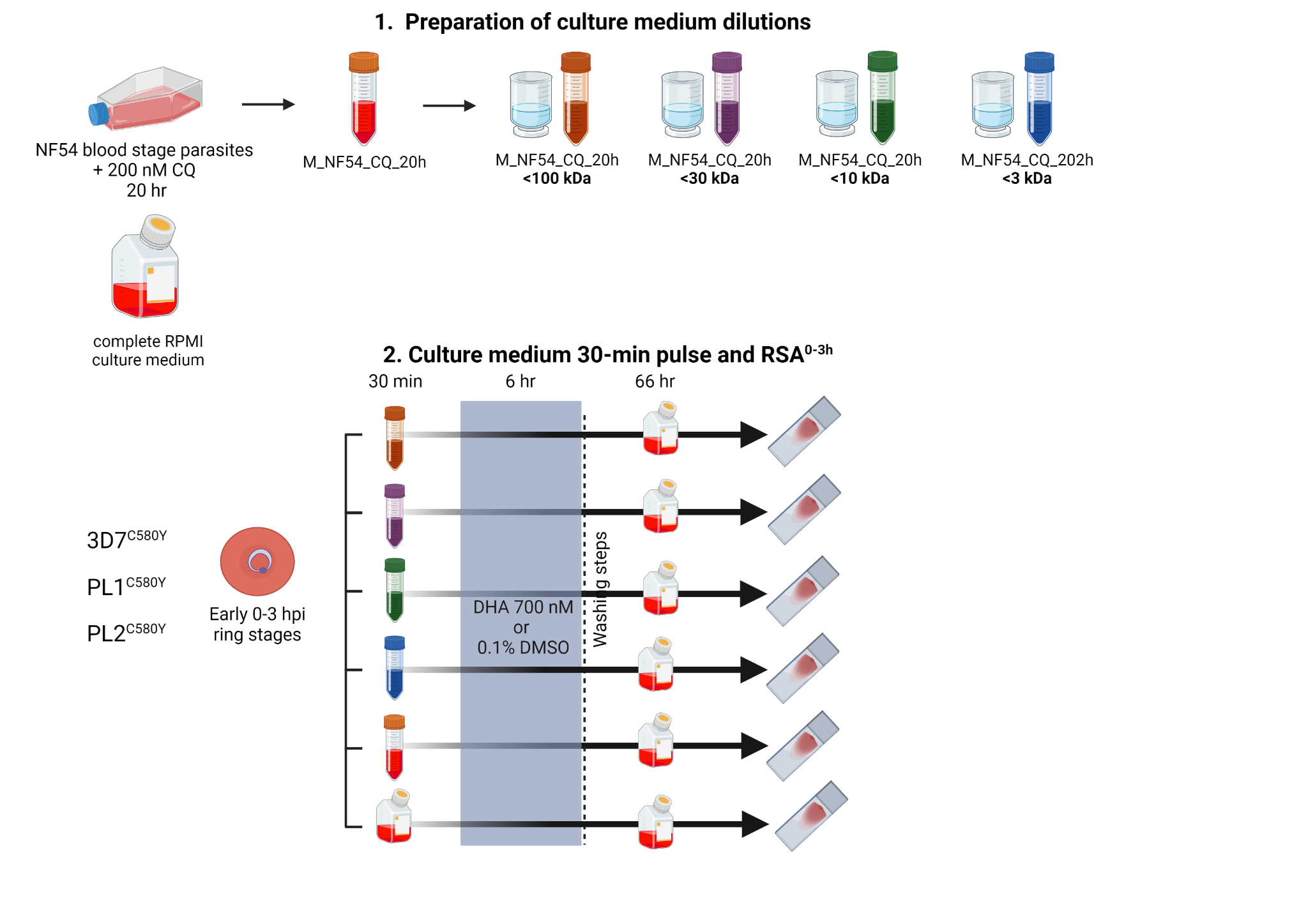


***Centricon® filtration.*** M_NF54_CQ20h medium was centrifuged (2000 rpm, 5 min), collected in a 50 mL tube, and stored at 37°C in a water bath. Centricon filters (Merck Millipore) were sterilised with 70% ethanol solution and prepared according to the manufacturer's instructions. We used four filter sizes to filter four independent M_NF54_CQ20h mediums. The filter pore sizes were as follows: 100 kDa (pore diameter: 10 nm), 30 kDa (pore diameter: 3 nm), 10 kDa (pore diameter: 1 nm) and 3 kDa (pore diameter: 0.3 nm). Centrifugations parameters and volumes were prepared according to the manufacturer's instructions.

Early 0-3 hpi PL2^C580Y^ ring-stages were then pre-exposed to complete RPMI medium (negative control), unfiltered M_NF54_CQ20h (positive control), and each of the four filtrates for 30 min before assessing their *in vitro* susceptibility to DHA using the RSA^0-3h^. All experiments were carried out in triplicate.

# **Mature-stage parasites treated with DHA release molecules into the culture medium that reduce in vitro susceptibility of ring-stage parasites to DHA.**

## ***Figure S6. Schematic representation of the experimental design.***


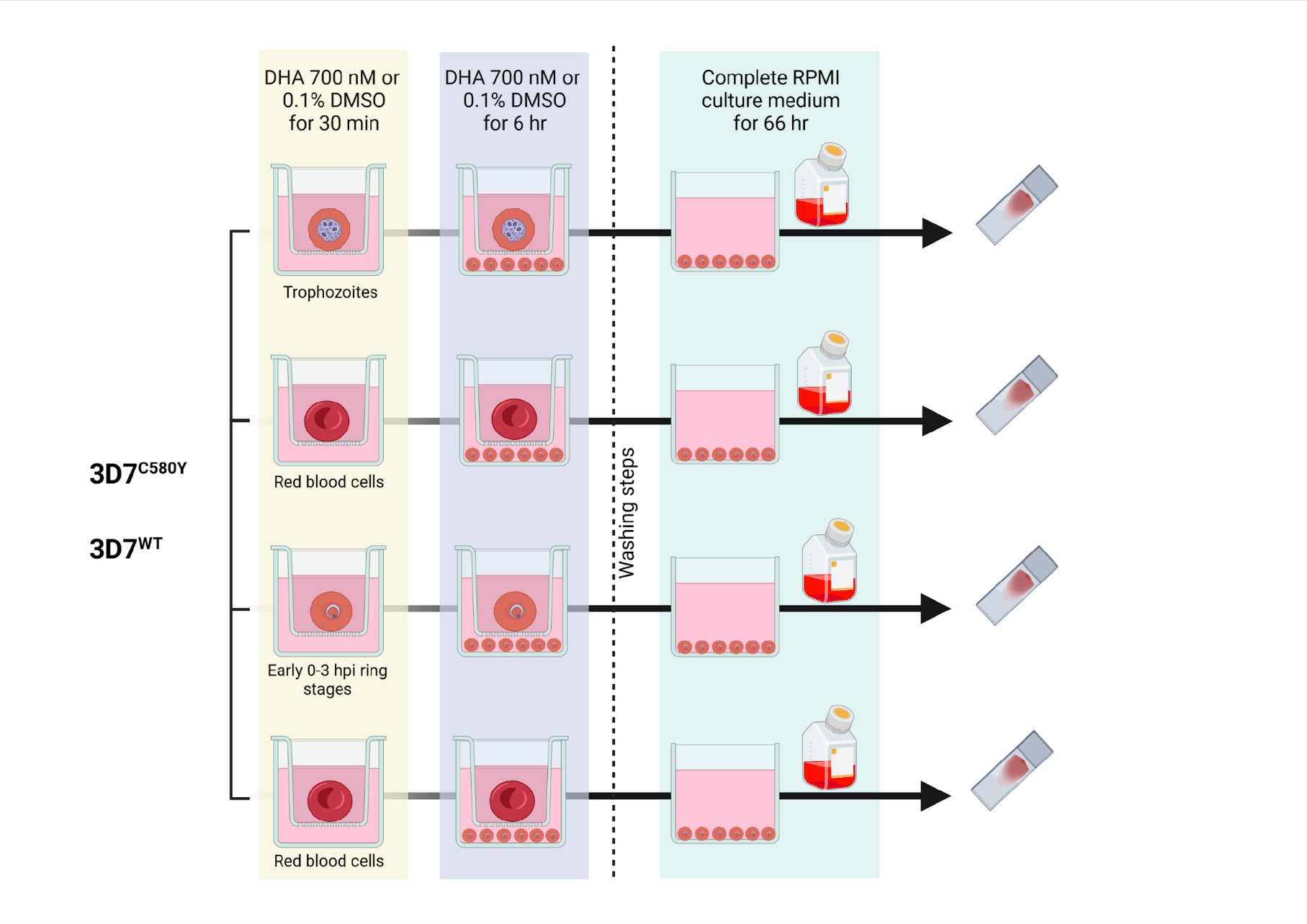


We first exposed the parasite samples (ring- or mature stages), or control uninfected RBCs, to 700 nM DHA or DMSO (0.1%) for 30 min in the upper chamber. We then seeded early 0-3 hpi ring-stages into the lower compartment and continued the culture for 6 hours in the same culture medium, allowing DHA and molecules released from the upper compartment to passively diffuse into the culture medium. Parasites in the lower compartment were collected, washed twice in incomplete RPMI medium to remove the drug, transferred, and cultured in complete RPMI medium for a further 66 hours. Parasitaemia was measured by microscopy after 72 hours. Parasite survival was expressed as the ratio of viable parasites in DHA to DMSO-treated samples, as previously described. All experiments were performed in triplicate.
